# Underwater Object Detection and Reconstruction Based on Active Single-Pixel Imaging and Super-Resolution Convolutional Neural Network

**DOI:** 10.3390/s21010313

**Published:** 2021-01-05

**Authors:** Mengdi Li, Anumol Mathai, Stephen L. H. Lau, Jian Wei Yam, Xiping Xu, Xin Wang

**Affiliations:** 1 College of Optoelectronic Engineering, Changchun University of Science and Technology, Changchun 130022, China; 2017100212@mails.cust.edu.cn; 2 School of Engineering, Monash University Malaysia, Jalan Lagoon Selatan, Bandar Sunway 47500, Malaysia; anumol.mathai@monash.edu (A.M.); stephen.lau@monash.edu (S.L.H.L.); Jian.yam@monash.edu (J.W.Y.); wang.xin@monash.edu (X.W.)

**Keywords:** single-pixel imaging, compressive sensing, super-resolution convolutional neural network

## Abstract

Due to medium scattering, absorption, and complex light interactions, capturing objects from the underwater environment has always been a difficult task. Single-pixel imaging (SPI) is an efficient imaging approach that can obtain spatial object information under low-light conditions. In this paper, we propose a single-pixel object inspection system for the underwater environment based on compressive sensing super-resolution convolutional neural network (CS-SRCNN). With the CS-SRCNN algorithm, image reconstruction can be achieved with 30% of the total pixels in the image. We also investigate the impact of compression ratios on underwater object SPI reconstruction performance. In addition, we analyzed the effect of peak signal to noise ratio (PSNR) and structural similarity index (SSIM) to determine the image quality of the reconstructed image. Our work is compared to the SPI system and SRCNN method to demonstrate its efficiency in capturing object results from an underwater environment. The PSNR and SSIM of the proposed method have increased to 35.44% and 73.07%, respectively. This work provides new insight into SPI applications and creates a better alternative for underwater optical object imaging to achieve good quality.

## 1. Introduction

In the underwater environment, reconstruction of objects is a challenging task, due to light attenuation by water absorption and the illumination volatility by the scattering medium [1,2]. Various approaches have been developed for imaging objects under low-light conditions and for improving the quality of underwater images. Polarimetric imaging systems [3,4] were used to reduce the backscattering effect and produced a good quality image. Although polarization correction can improve the renovation quality, it also blocks part of the light incident on the detector, thereby reducing the signal to noise ratio and complicating the problem of imaging underwater. Range-gated imaging was developed in Mariani et al. [5] by combining the time of flight technique. The use of a pulsed laser as a light source enabled the authors to measure the distance of the object from the light source, thereby calculating the depth of the object by eliminating the backscattering effect. However, relative to other cameras, range-gated systems are generally more expensive, more complicated to operate, and limited in resolution and frame rate. Geotagging and color correction approaches were implemented to successfully generate 2D and 3D images of the underwater environment [6]. To capture underwater images, a digital camera with waterproof housing was used and the location of the camera was identified through geo-tagging. Though it could produce good-quality 2D and 3D maps of the underwater environment, requirements such as clear water and calm surface conditions limited its performance. To further improve the underwater image quality, in Lu et al. [7] used a multi-scale cycle generative adversarial network. In that, the discriminator could produce high-quality images under homogeneous lighting conditions. However, it is failed under inhomogeneous illumination. The work was extended in Tang et al. [8] using an algorithm called dark channel prior to further improving underwater image quality. Empirical mode decomposition was developed in Çelebi et al. [9], where obtained underwater images were decomposed based on the spectral components and then, again, constructed by combining the intrinsic mode functions to enhance visual image quality. However, this three-channel component calculation method increased the amount of calculation. Most of the schemes utilize the silicon-based Charge Coupled Device (CCD) or Complementary Metal Oxide Semiconductor (CMOS) to image underwater objects. The limited response of the detection medium to a specific bandwidth has always been a drawback of those sensors imaging systems under low illumination conditions.

Recently, single-pixel imaging (SPI) has attracted widespread attention in imaging objects under low-light conditions and a scattering medium. SPI uses pre-programmed modulated light patterns and knowledge of the scene under view to acquire spatial information on the target [10,11]. In projection-based SPI, the object is illuminated with two-dimensional spatially coded patterns and collects reflected or transmitted light signals from the object with the single-pixel detector (SPD) to gather the fine details of the object [11]. In addition to cost-effectiveness, other advantages of SPI are low dark current and light sensitivity, producing good-quality images. Simultaneously, the introduction of compressed sensing (CS) in SPI has enabled reconstruction with fewer measurements [12,13]. Owing to these improvements, SPI has been exploited in scattering media [14,15]. Although it has been proved that SPI can be imaged underwater, the fluctuation in illumination caused by absorption and the scattering medium of water still seriously reduce the reconstruction quality. To suppress these effects. Chen, et al. [16] proposed a SPI detection scheme in which an object can be recovered through turbid water by transmission signal data. Although it has been proved that SPI can be imaged underwater, the scattering and absorption of water seriously reduce the reconstruction quality. In addition, these methods also have some noise on the detector, decreasing the image quality [17]. Hence, for SPI underwater, an SPI restoration algorithm that is robust to noise is demanded. Moreover, limitations in the visual aspect of the above imaging system can be overcome by combining both deep learning and a basic SPI system.

Deep learning [18], as an emerging field of research, has proven its performance in image processing, such as image recognition [19,20], object detection [21], and person pose estimation [22]. When combining neural networks with SPI systems, there are two ways to get the final target image. One exploits the robust neural network to rehabilitate the object image and the other trains and predicts images with the neural network after reconstruction. For the former, Higham, et al. [23] utilized MATCONVNET to create a deep convolutional auto-encoder using the patterns as the encoding layer and Hadamard patterns as the optimization basis. For subsequent decoding layers, three conventional layers were used to recover the real-time high-resolution video. For the latter scheme, Rizvi, et al. [24] raised a deep convolutional autoencoder network that uses reconstructed under-sampled training and testing images as input and high-quality predicted images as output. Compared with previous methods, deep learning algorithms have been proved to be more reliable for restoration. Therefore, the employment of deep learning in SPI system has brought drastic changes in image quality.

Though different studies about the influence of turbulence [25] and deep learning on SPI are discussed in Dutta et al. [26] and Jauregui-Sánchez et al. [27], there are limited studies that focus on SPI with deep learning for underwater image reconstruction. Therefore, in this paper, we leverage single-pixel imaging and deep learning to improve underwater imaging restoration. An experimental setup was established to verify the presented method. The effectiveness of our technique is demonstrated through both experiments and simulation. We summarize them as follows:

- An optical imaging system based on SPI is developed for imaging objects in the underwater environment. Our experimental results validated that the recovered object by the underwater SPI system is affected by scattering and absorptions.
- Compressive sensing-based super-resolution convolutional neural network (CS-SRCNN) is implemented by combining the advantages of the SRCNN and SPI system. The newly introduced CS-SRCNN takes reconstructed underwater SPI images to train the network and to predict high-resolution images.
- We also demonstrate the effectiveness of our technique through simulation. In the simulation, the proposed method can restore objects with a low sampling rate and can produce more robust reconstructions. Our experimental results also validated that the recovered object by the underwater SPI system is affected by scattering and absorptions.
- We experimentally demonstrated reconstruction to get better results with a low sampling rate of only 30%.

## 2. Underwater Object Reconstruction

### 2.1. Theory

#### 2.1.1. Compressive Sensing

**Figure 1:**
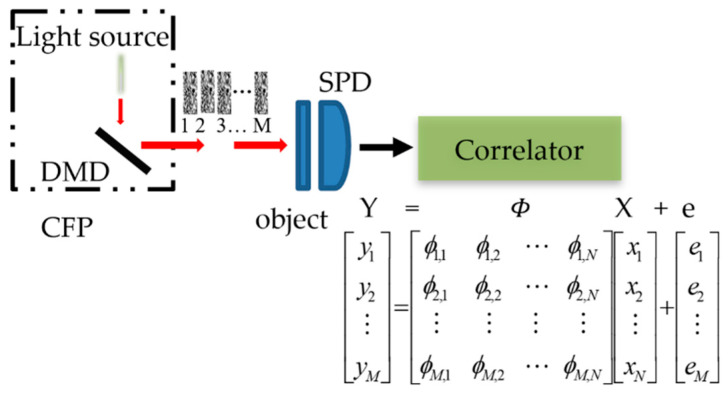
The single-pixel imaging setup. DMD: Digital Micromirror Device, CFP: Calculated Filed Pattern, and SPD: Single-Pixel Detector.

Figure 1 shows the schematic diagram of the SPI system. Assuming that *X* is the column vector reshaped from the *N* = *p* × *q* pixel image and that it is sparsely sampled by measurement matrix Φ, the corresponding measurement data can be expressed as follows:

(1) Y=ΦX+e

where Y is the measurement data by stacking of $y_{i}$, which is an *M*x*1* column vector having linear measurements. *M* is the number of measurements used for image acquisition. The measurement matrix $\Phi \in {\mathbb{R}}^{M\times N}$ contains *M* row vectors, which are the stacking of the 2-dimension coded patterns $\phi_{m}$. e of size *M*x*1* denotes the noise. Equation (1) can be written as follows:

(2) $$\left[\begin{array}{c} y_{1}\\ y_{2}\\ \vdots \\ y_{M} \end{array}\right]=\left[\begin{array}{cccc} \phi_{1,1} & \phi_{1,2} & \cdots & \phi_{M,N}\\ \phi_{2,1} & \phi_{2,2} & \cdots & \phi_{M,N}\\ \vdots & \vdots & \vdots & \vdots \\ \phi_{M,1} & \phi_{M,2} & \cdots & \phi_{M,N} \end{array}\right]\left[\begin{array}{c} x_{1}\\ x_{2}\\ \vdots \\ x_{N} \end{array}\right]+\left[\begin{array}{c} e_{1}\\ e_{2}\\ \vdots \\ e_{M} \end{array}\right]$$

A measurement matrix Φ was constructed with a random pattern combined with the observed signal $y_{i}$ to obtain a system of equations. However, as mentioned above, Φ has fewer rows than columns (*N* = *p* × *q*). Therefore, solving out *X* using Y and Φ seems impossible because the solution of the system of equations is not unique. To address the issue, we relied on the principle of compressed sensing (CS) [28], which can be used effectively for sparse images. Sparsity indicates that a signal is represented in an appropriate basis, where most of the information in images are close to zero. In most instances, the gradient integral of a natural signal is statistically low; hence the image can be compressed by the CS method. When *X* is k-sparse, it can be recovered with M=O(klogN) incoherent nonadaptive linear measurements. Equation (1) can be summarized as follows:

Let $D_{i}x$ denote the discrete gradient vector of *X* at position (*i*,*j*), D be the gradient (horizontal $D_{i}^{h}x$ and vertical $D_{i}^{\nu }x$) operator with the following operational definitions:

(3) $$D_{i}^{h}x=\{\begin{array}{l} x_{i+1,j}-x_{i,j},\;1\leq i<M\\ 0,i=M \end{array}$$

(4) $$D_{j}^{v}x=\{\begin{array}{l} x_{i,j+1}-x_{i,j},\;1\leq j<N\\ 0,j=N \end{array}$$

The total variation regularization (TV) in *X* is simply the sum of the magnitudes of this discrete gradient at each point and the error term:

(5) $${\left\|x\right\|}_{TV}=\sum_{i,j}\sqrt{{\left(x_{i+1,j}-x_{i,j}\right)}^{2}+{\left(x_{i,j+1}-x_{i,j}\right)}^{2}}$$

(6) $$TV(X)=\sum_{i}{\left\|D_{i}x\right\|}_{1}+\frac{\mu }{2}{\left\|Y-\Phi X\right\|}_{2}^{2}$$

where $\sum_{i}{\left\|D_{i}x\right\|}_{1}$ is the discrete TV of *X*, μ is a constant scalar used to balance these two terms, and ${\left\|x\right\|}_{1}={\sum_{i=1}^{N}\left|x_{i}\right|}^{1}$ represents $\ell_{1}$ norm. TV regularization to recover the image is discussed in Li [29]. The first term in Equation (6) is small when *D_i_x* is sparse. When the optimal *X* is consistent with Equation (1) with a small error, the second term is small [30]. The image reconstruction problem of Equation (1) can be expressed as follows:

(7) min TV(X) subject to ΦX=Y

Accurate recovery can be achieved by solving a convex optimization program that is easy to handle [31].

#### 2.1.2. The Super-Resolution Convolutional Neural Network

Convolutional neural network (CNN), a typical deep learning algorithm, has been widely used in image processing due to its powerful feature learning function in computer vision research. SRCNN [32,33] is the first end-to-end super-resolution algorithm using the CNN structure. An SRCNN architecture was adopted as the benchmark, which learns to map between low or high-resolution images. The low-resolution (LR) components, *X*_SPI_, were loaded into bicubic interpolation to get interpolated components *X*_II_. The patch extraction layer extracted patches from the bicubic interpolated components, and feature extraction by convolution can be denoted as follows:

(8) $$F_{1}=\max(0,W_{1}*X_{II}+B_{1})$$

where the variables *F*, *X*_II_, *W*_1_, and *B*_1_ represent the mapping function, the original high-resolution (HR) image after interpolation, the filters, and the biases (*n*_1_-dimensional vector), respectively, and * represents the convolution operation. The size of *W*_1_ is *n*_1_ × *c × f*_1_ × *f*_1_, *f* is the size of the filter, c is the number of channels contained in the input image, and n is the number of convolution kernels, with suffix 1 indicating the first layer. A Rectified Linear Unit (ReLU) was used as the activation function of the network. The function expression of ReLU is as follows:

(9) ReLU=max(0,x)

ReLU can be computed faster than traditional activation functions such as sigmoid and allows for easier optimization of the neural network. The nonlinear mapping layer maps from LR space to HR space. The operation is as follows:

(10) $$F_{2}=\max(0,W_{2}*F_{1}+B_{2})$$

The size of *W*_2_ is *n_2_* × *n*_1_ × *f*_2_ × *f*_2_, with *B_2_* being an n_2_-dimensional vector. This layer outputs the *n_2_*-dimensional feature map as an input to the third layer.

The reconstruction of HR images in the third layer can be expressed as follows:

(11) $$F_{3}=\max(0,W_{3}*F_{2}+B_{3})$$

where the size of *W*_3_ is *c* × *n_2_* × *f*_3_ × *f*_3_. Combining these three operations constitutes a CNN in which all filter weights and deviations are optimized. In addition, during the training phase, the mapping function F needs to estimate the network parameters $\Theta =\{W_{1},W_{2},W_{3},B_{1},B_{2},B_{3}\}$. The reconstructed images were described as $F(X_{II};\Theta )$, and the HR image was *X_SR_*. The error between $F(X_{II};\Theta )$ and the real image *X_SR_* was minimized. To achieve this goal, the mean square error (MSE) was chosen to construct the loss function of the network model of this paper. The loss function L is expressed as follows:

(12) $$L(\Theta )=\frac{1}{o}{\sum_{i=1}^{O}\left\|F(X_{IIi};\Theta )-X_{SRi}\right\|}^{2}$$

where *o* is the number of training images and *X_SRi_* is a set of HR image. The loss function was minimized by applying the gradient descent method and the standard back-propagation algorithm.

### 2.2. The Reconstruction of Underwater Single-Pixel Imaging based on Compressive Sensing and Super-Resolution Convolutional Neural Network

**Figure 2:**
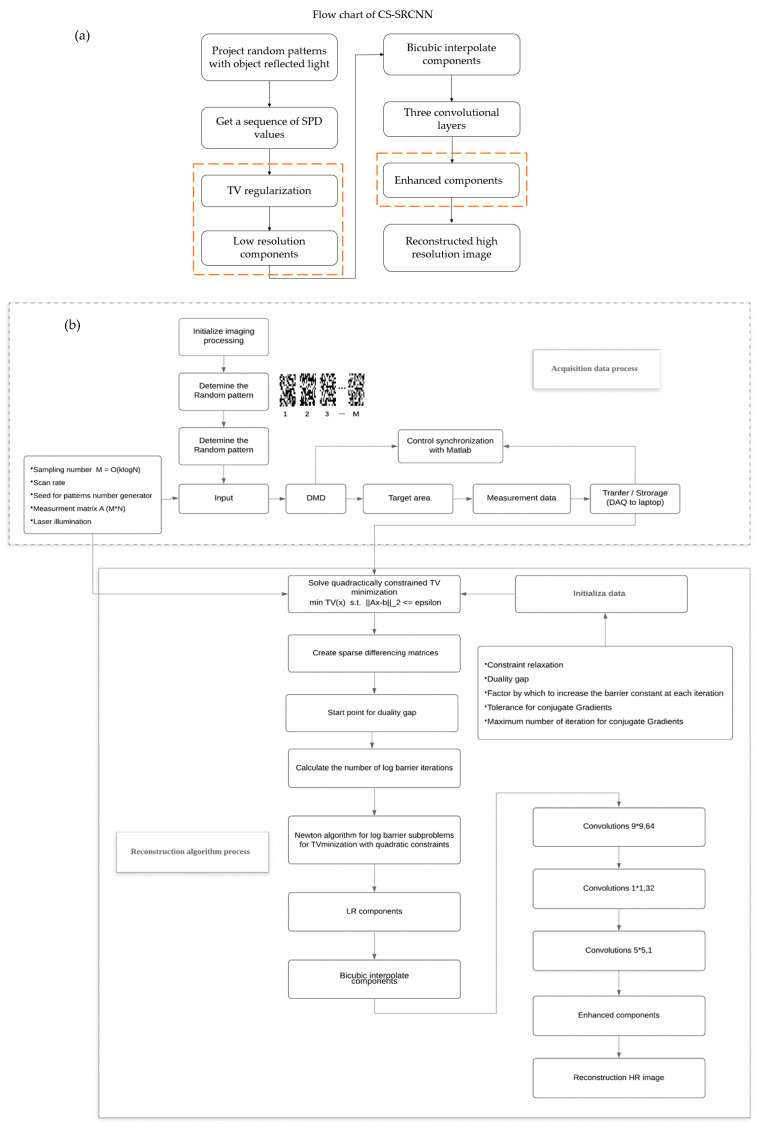
Activity diagram of the proposed reconstruction algorithm: (**a**) traditional single-pixel imaging and super-resolution convolutional neural network (SRCNN), and (**b**) the proposed method in this paper.

According to those theories, we regarded CS image regeneration as an inverse problem and tackled this question based on SRCNN. In this study, we propose an underwater object reconstruction CS-SRCNN method which combines the improved SRCNN and SPI system. In addition, the specific implementation architecture of the proposed method is shown in Figure 2. This method architecture was adopted as the benchmark, which learns to map between low or high-resolution images. That is, high-frequency components were increased in low-resolution (LR) components. In the mapping process, network measurement data were taken as input and produce high-resolution (HR) images were taken as output [34,35].

**Figure 3:**
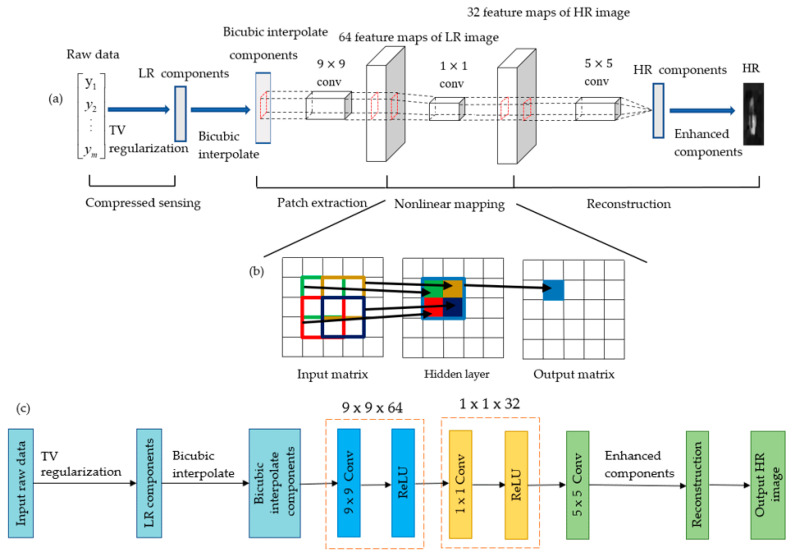
Architecture of the proposed reconstruction: (**a**) architecture of the proposed reconstruction; (**b**) convolved data from the input matrix to the output matrix, where the convolution process moves 1 step in the input matrix; and (**c**) a schematic diagram of our method.

The architecture combined both deep learning and traditional sparse coding technique ( in Figure 3). The CS-SRCNN can be divided into their respective functions, namely patch extraction, nonlinear mapping, and final reconstruction. The first part of the SRCNN (patch extraction) consisted of a CS layer and a three-layer CNN. The input to the entire neural network was M-dimensional compressed raw data from SPI system. The raw data ran iterative processing by TV regularization to attain LR components. The network enlarged the LR components by bicubic interpolation, which is a preprocessing step for CNN. Compared to other methods, bicubic interpolation is faster and does not introduce too much additional information. After that, the first convolutional layer extracted 9 × 9 LR patches. The output was passed to the rectified linear unit (ReLU) activation function. The second part of the SRCNN (nonlinear mapping) made use of a convolution layer with a kernel size of 1 × 1 to output 64 feature maps, which were then concatenated into a matrix. The output was passed to the ReLU activation function. In the third part of the SRCNN (reconstruction), the matrix from the preceding layer was passed through a convolution layer with a kernel size of 5 × 5. HR feature vectors were reconstructed, and all HR patches were combined to form the highest enough HR components. Subsequently, we applied postprocessing (e.g., filtering to remove noise) to the reconstructed images.

In our study, the dataset was divided into two subsets: the training set and test set. We used 400 images of handwritten digits downloaded from the MNIST database with the corresponding SPI images to train the SRCNN. The other 100 images were put into the test set. MNIST handwritten digits (28 × 28 resolution) were resized into the correct resolution (e.g., 32 × 32). For each training image, the same set of M different random patterns was applied in both the simulation and experiment (more details in Section 3). After the set of M patterns was run with the object, we attained a light intensity signal *y_i_* of length M. We then paired up the resulting signal *y_i_* and the corresponding 2D image. The objects were replaced, and the above simulation process was rerun to obtain another pair of labeled data. For training, no additional noise was added to the simulated light intensity signal. This process was run repeatedly across the whole 400 images in the training set and were paired up with *y_i_*.

## 3. Simulation Results and Analysis

**Figure 4:**
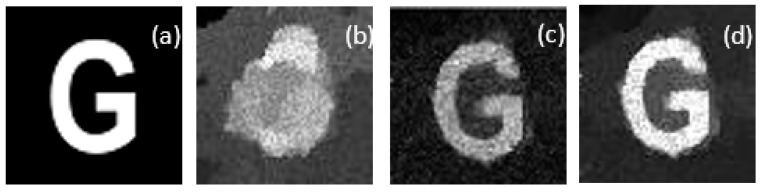
The images of different compression ratios that were reconstructed: (**a**) an original target image with pixels size 32 × 32 and (**b**–**d**) reconstructed images using different numbers of random speckle patterns (the M takes the values 50, 200, and 300, respectively, and different compression ratios from 4.88% and 19.53% to 29.29%).

In the underwater SPI system, to investigate the effect of the number of measurements and water turbidity, random patterns were employed to sample the object with 32 × 32 resolution. The simulation method using Gaussian blur [36] (different Gaussian noises of 10, 15, and 25 dB were added to simulate different underwater turbidity conditions) as the disturbance factor simulated scattering of the water medium and qualitatively analyzed the non-disturbance ability of SPI under different sampling rates in turbid water. The simulation results for different sampling rates are shown in Figure 4. We can see that there are blurry artifacts in the refurbished images at different sampling rates between 4.8–30%.

**Figure 5:**
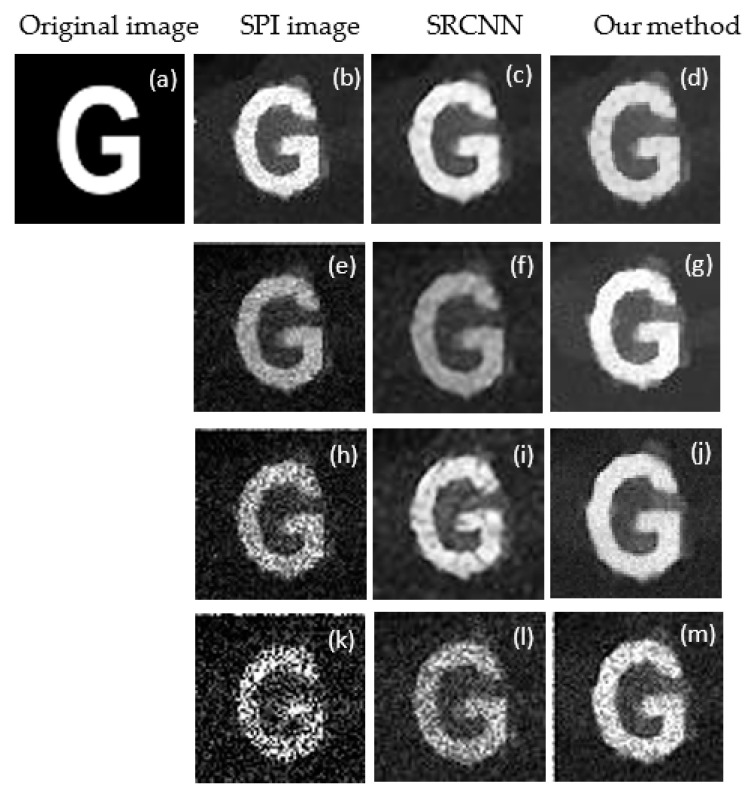
Image reconstruction results at different turbidities using random patterns in clear water under 300 measurements: (**a**) original image; (**b**–**d**) single-pixel imaging (SPI) image, SRCNN image, and our method image with 0 NTU; (**e**–**f**) SPI image, SRCNN image, and our method image with 20 NTU; (**d**) SPI image, SRCNN image, and our method image with 40 NTU; and (**k**–**m**) SPI image, SRCNN image, and our method image with 60 NTU.

Furthermore, to inspect how the image quality changes in the underwater environment, we simulated CS-SRCNN reconstruction for an image of the letter G with a sampling rate of 0.29 for different underwater turbidities. We then verified the influence of turbidity on the reconstruction quality by applying the conventional SRCNN and our method. According to our setup, we compared images from SPI, SRCNN, and our method by applying upscaling factors of 3 on the underwater image. Figure 5 shows the visual effects at different turbidities (0, 20, 40, and 60 nephelometry turbidity unit (NTU)). As the turbidity increased from 0 NTU to 60 NTU, we observed a significant degradation of the reconstructed image, comparing Figure 5b with Figure 5e–g. However, CS-SRCNN can reconstruct clear images, even if the turbidity is as high as 60 NTU, and the reconstructed image can still be satisfactory with a sampling ratio of about 29%.

**Figure 6:**
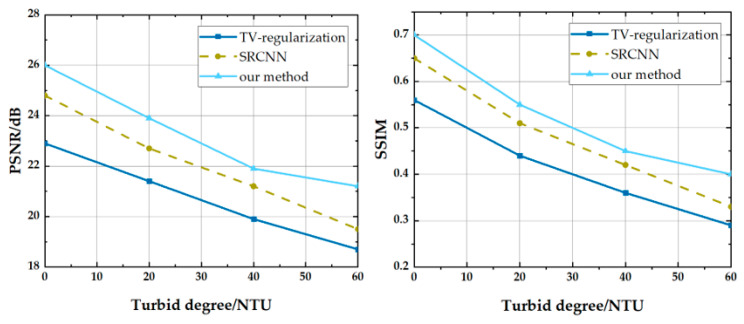
The index values for Bicubic and SRCNN at different magnifications.

Figure 6 compares the peak signal to noise ratio (PSNR) and structural similarity index (SSIM) of images taken with different turbidities of SPI before reconstructed with TV-regularization, SRCNN, and CS-SRCNN. We kept the number of measurements constant at 300. The figure shows that both PSNR and SSIM decreased with turbidity for all three methods of reconstruction. In the low turbidity region from 0 NTU to 40 NTU, the PSNR and SSIM of the three methods declined at a similar rate. On the contrary, in areas where the turbidity is higher than 40 NTU, the imaging quality of our method shows robustness (the slope tends to be flat) while the other two approaches followed the former trend. Since these three evaluation factors have good robustness to the influence of the scattering factor from the water medium, this makes CS-SRCNN more sensitive, and the results of underwater imaging can be further enhanced.

We also compared the image recovered by our method with the images reconstructed by the end-to-end learning method and image processing method, which is the scattering media image recovery method based on a polarimetric ghost imaging system formulated by Li, et al. [37]. The PSNR and SSIM were used to evaluate and compare the results. The results of those methods are shown in Table 1. Compared with Li et al.’s method, the reconstructed image PSNRs and SSIMs in our method were significantly increased by 17.39%/83.78% and 16.11%/90.32%, which means that our method has better performance than the other methods.

## 4. Experimental Results and Analysis

### 4.1. Experimental Setup

**Figure 7:**
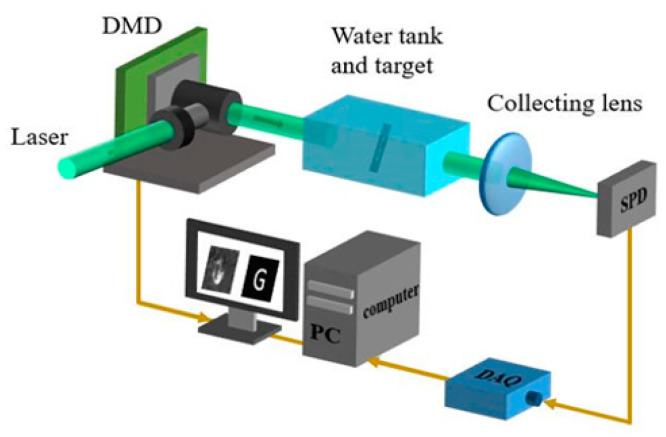
Schematic diagram of the underwater object detection system.

The experimental setup for the underwater SPI system is illustrated in Figure 7, which consists of a laser source and a DMD to project binary patterns. A fast response time SPD with a collection lens was used to measure the transmission intensity resulting from each pattern. The corresponding intensity values were captured by a data acquisition (DAQ) device. The object image was acquired by correlating the values from the DAQ with the pattern martrix.

**Figure 8:**
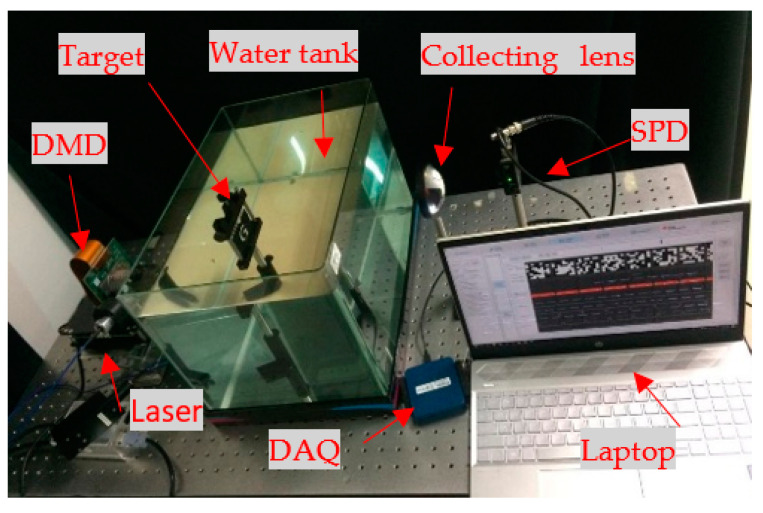
Experimental setup implemented in our lab environment.

The experimental setup is shown in Figure 8. The continuum laser is monochromatic, highly intense, and power-efficient and produces a coherent wavelength of 520 nm (the laser power was 20 mW). It is, therefore, a viable choice of light source that illuminates the DMD. The DMD allows for a configurable input (MATLAB programming and light source)/output (random pattern) trigger for convenient synchronization with continuum laser and SPD peripheral devices. The DLP (DLP Light Crafter 6500, Texas Instruments) provides a true HD resolution of 1920 × 1080, and more than 2 million programmable micromirrors by the DMD chip mounted on it. The pre-programmed random patterns are produced by the DMD, which is in the form of a 32 × 32 matrix. Each light pattern that passed through the underwater object (transparent “G”; the length, width, and height of the water tank were 20 cm, 20 cm, and 25 cm) was directed to a collecting lens (the model used was THORLABS LMR75/M). The collecting lens focused the light on the SPD (the model used was PDA36A2), which acted as a bucket detector and had a bandwidth of 12 MHZ and an area of 13 × 13 mm^2^. The total light intensity value detected by the digital-to-analog converter (National Instrument DAQ USB-6001) was converted to an electrical signal and was transmitted to a laptop via USB for image reconstruction. The synchronization between DMD and SPD was done by sending a trigger signal to the DMD to refresh the patterns. Simultaneously, the trigger signal was sent to the DAQ to attain the SPD voltage. The data management and sampling were controlled and synchronized by MATLAB software. The automation scripts for data collection were executed with the MATLAB software on a laptop. After minimizing the total variation, the reconstructed algorithm was implemented to reconstruct the image.

### 4.2. TV Regularization (Compressive Sensing) Reconstruction

**Figure 9:**
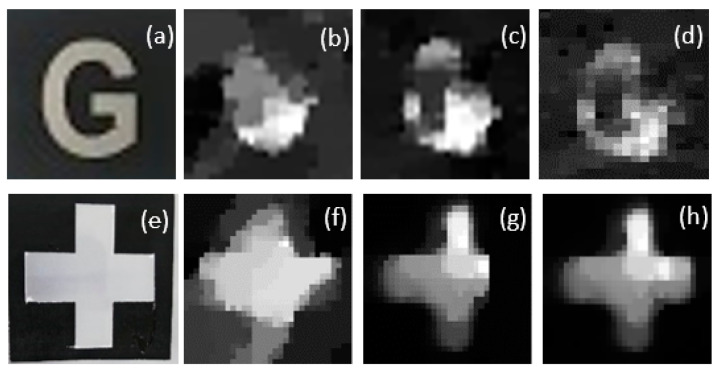
Images of different compression ratios reconstructed: (**a**) an original target image with pixel size 32 × 32; (**b**–**d**) reconstructed images using different numbers of random speckle patterns (M takes the values 100, 200, and 300, respectively, and different compression ratios from 9.76% and 19.53% to 29.29%); (**e**) an original target image with pixel size 32 × 32; and (**f**–**h**) reconstructed images using different numbers of random speckle patterns.

The experimental results of the reconstruction of underwater SPI using TV regularization are shown in Figure 9. The reconstruction was affected by the number of measurements, imaging speed, and compressibility. Random patterns were employed to reconstruct the object with 32 × 32 resolution. To investigate the effect of the number of measurements on the underwater image, different numbers of measurements were used to capture and reconstruct the images.

Also, Figure 9 represents the reconstruction results for three different numbers of random patterns. The images in Figure 9b–d,f–h represent the reconstruction results for 100, 200, and 300 random patterns, respectively. It can be seen that the simple object (“+”) was restored better than the complex object (“G”). Furthermore, the object can be reconstructed even with 29% of the total number of pixels in the original image by using CS.

In order to quantify the accuracy of image reconstruction results, image quality assessment techniques such as the mean square error (MSE), PSNR, SSIM, and visibility (V) were employed. The MSE was calculated by comparing the reconstructed image and original object image, and it is defined as follows:

(13) $$MSE=\frac{1}{pq}\sum_{i=0}^{p-1}\sum_{j=0}^{q-1}{\left[x(i,j)-X_{R}(i,j)\right]}^{2}$$

where *x* is the original image with *p* x *q* resolution, and *X_R_* is the reconstructed image. Suppose $x_{\max}^{2}=2^{K}-1$, where *K* represents the number of bits used for a pixel, *K* = 8, and *x_max_* = 225. Similarly, PSNR describes the ratio of original image pixels to reconstructed pixels, and it is defined as follows:

(14) $$PSNR(dB)=10\mathrm{lg}\frac{x_{\max}^{2}}{MSE}=10\mathrm{lg}\left[\frac{x_{\max}^{2}pq}{\sum_{i=0}^{p-1}\sum_{j=0}^{q-1}{\left[x(i,j)-X_{R}(i,j)\right]}^{2}}\right](dB)$$

SSIM is a full-reference metric that describes the statistical similarity between two images. The indicator was first proposed by the University of Texas at Austin’s Laboratory for Image and Video Engineering [38]. This is given by the following:

(15) $$SSIM=\frac{\left(2\mu_{x}\mu_{X_{R}}+c_{1})(2\sigma_{xX_{R}}+c_{2}\right)}{\left(\mu_{x}^{2}+\mu_{X_{R}}^{2}+c_{1})(\sigma_{x}^{2}+\sigma_{X_{R}}^{2}+c_{2}\right)}$$

where $\mu_{x}$ and $\mu_{X_{R}}$ are the averages of *x* and *X_R_*, respectively; $\sigma_{x}$ and $\sigma_{X_{R}}$ are standard deviations of *x;* X and $\sigma_{xX_{R}}$ are covariances of *x* and *X_R_*, respectively; c_1_ and c_2_ are variables to stabilize the division with the weak denominator (constants); *c*_1_ *= (K*_1_*L)*^2^; and *c*_2_ *= (K*_2_*L)*^2^. Generally, *K*_1_ = 0.01, *K*_2_ = 0.03, and *L* = 255 (*L* is the dynamic range of the pixel value, generally taken as 255). The visibility (V) is defined as follows:

(16) $$V=\frac{\langle S_{in}\rangle -\langle S_{out}\rangle }{\langle S_{in}\rangle +\langle S_{out}\rangle }$$

where <*S_in_*> and <*S_out_*> are the average values of SPI of the interesting region and the background region, respectively. The PSNR, SSIM, and V were calculated using the MATLAB function.

As can be seen from Table 2 and Table 3, the measurements are directly proportional to the PSNR and SSIM. To be clear, the PSNR and SSIM ranges are higher for image restoration at more measurements. For example, in Table 2, PSNR = 11.59 dB, SSIM = 0.26, and V = 0.29 for 300 measurements and PSNR = 9.02dB, SSIM = 0.10, and V = 0.24 for 100 measurements. Compared with the third column, the reconstructed images PSNR, SSIM, and V in the first column were significantly increased by 28.49%/1.6/20.83%. Therefore, the more the number of measurements, the better the similarity of images reconstruction.

### 4.3. Reconstruction Based on Compressive Sensing Super-resolution Convolutional Network (CS-SRCNN)

**Figure 10:**
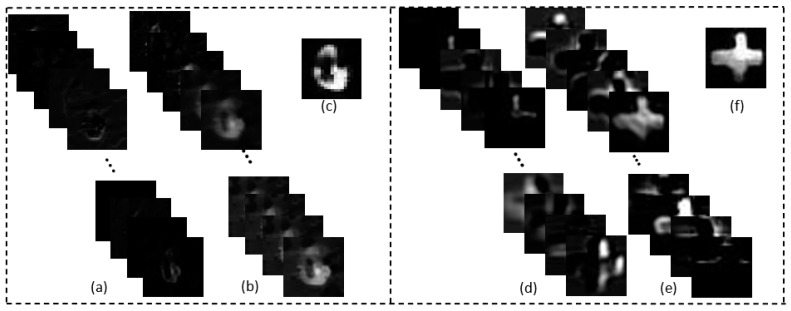
The results of each layer of convolution: (**a**,**d**) part of the first 64 feature maps, (**b**,**e**) part of the second layer of 32 feature maps, and (**c**,**f**) part of the 1-feature map of the third layer.

In this scheme, the data set with 300 measurements from an underwater SPI system was fed into the TV regularization algorithm. The resultant output enlarged the desired matrix size by bicubic interpolation. The interpolated components were set as the input to the following layers. The output of each layer of convolution is shown in Figure 10. For the first layer, the convolution kernel size was 9 × 9 (*f*_1_ × *f*_1_), the number of convolution kernels was *n*_1_ = 64, and the output was 64 feature maps. Optimization of the output was performed by ReLU as a nonlinear activation function. For the second layer, the convolution kernel size was 1 × 1 (*f*_2_ × *f*_2_), the number of convolution kernels was *n_2_* = 32, and the output was 32 feature maps. Similarly, for the third layer, the convolution kernel size was 5 × 5 (*f*_3_ × *f*_3_), the number of convolution kernels was *n_3_* = 1, and the output was 1 feature map. The final feature map is the reconstructed HR components.

**Figure 11:**
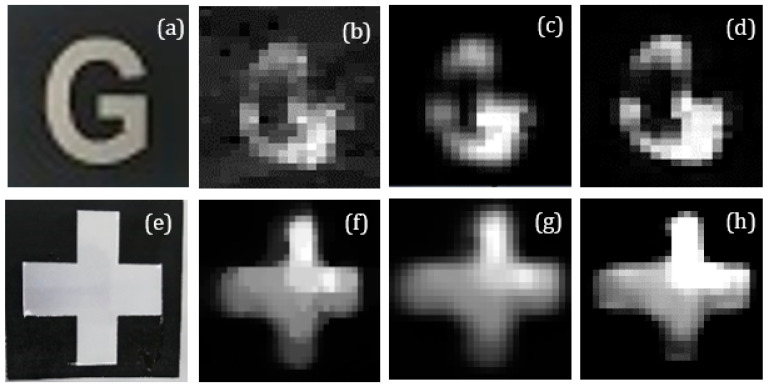
Image reconstruction results at different methods using random patterns in clear water under 300 measurements: (**a**,**e**) original image, (**b**,**f**) SPI image, (**c**,**g**) SRCNN image, and (**d**,**h**) our method image.

Furthermore, in this part, according to our setup, a comparison was made for SPI, SRCNN, and our method images by applying upscaling factors of 3 on the underwater image. Figure 11 shows the visual effects of different methods.

To investigate the relationship among the image quality of super-resolution images reconstructed by the CS-SRCNN scheme and SPI image, two indicators are commonly used such as PSNR and SSIM. The analysis of results focused more on the complex letter “G”. The higher PSNR and SSIM, the closer the pixel value to the standard. With the idea of controlling the variates, the index values for SRCNN and our method at upscaling factors of 3 are presented in Table 4 and Table 5 by keeping other conditions such as compression ratio of the reconstructed image, the reconstructed image algorithm, and so on constant. Moreover, the respective PSNR and SSIM for each recovered image in Figure 9d,h are shown in Table 4 and Table 5. Compared with the third column, the reconstructed images PSNR, SSIM, and V in the second column increased significantly by 29.83%/40.62%/10.00%, and 15.97%/44.00%/6.45%, respectively. We can know that PSNR, SSIM, and V have been significantly improved, consistent with imaging theory. The results validate our analysis of neural networks in underwater SPI.

**Figure 12:**
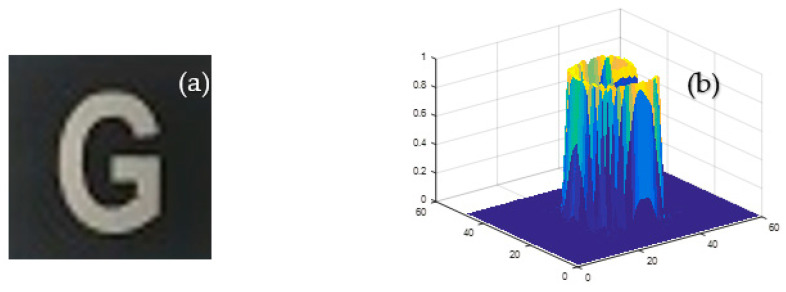
Test image: (**a**) original image and (**b**) the three-dimensional surface of the original image.

The above analysis only evaluates the quality of images from the two aspects PSNR and SSIM. To comprehensively evaluate the performance of the algorithm, a comparative analysis was performed using the original image shown in Figure 12a at different compression ratios for TV regularization and our algorithms.

**Figure 13:**
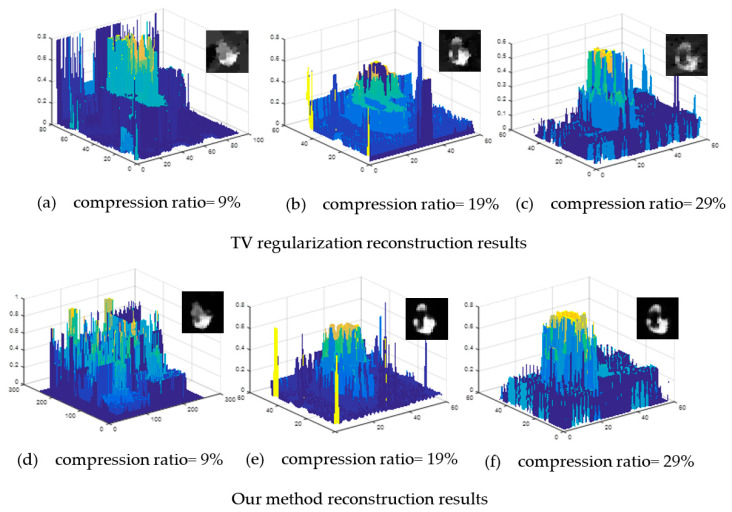
Reconstruction algorithm comparisons: (**a**–**c**) TV regularization reconstruction results at different compression ratios and (**d**–**f**) our method reconstruction results at different compression ratios.

In comparison, the images with compression ratios of 0.70, 0.13, and 0.20 were reconstructed. Figure 13a–c shows the reconstruction results for TV regularization for different compression ratios. From the figure, the signal reconstructed is closer to the original image signal when compression ratio increases and convergence occurs. Figure 13d–f are the recovery results corresponding to our reconstruction algorithm. As the compression rate increases, the reconstruction accuracy of the image gets higher and higher; however, the result shows that there are peaks, which are quite different from the original signal distribution curve. The main reason for this is that our reconstruction algorithm performs block processing on the input image and then reconstructs each small block independently and combines it into the whole picture. Therefore, there is an image blocking artifact problem which is not obvious when the compression ratio is high but is more evident at a low compression ratio. Filtering and smoothing can be performed at the end of reconstruction to alleviate the blocking artifact problem in the resultant HR image.

## 5. Conclusions

In the current study, an improved underwater SPI system and an image super-resolution method are proposed, which are cost-effective for underwater imaging. This system can overcome the limitations of obtaining good-quality images in low-light conditions. The proposed SPI system employed a combination of the CS algorithm and a neural network to enhance the quality of the images to reduce sampling time and postprocessing of data. Referring to the network structure of an CS-SRCNN, the employed network algorithm successfully deduced the HR images from being trained with multiple measurement data. The resultant 2D underwater image using the proposed single-pixel imaging setup and network algorithm ensures better quality compared to the conventional SRCNN method. The experimental results show that, while the PSNR and SSIM of SPI images reach 11.57 dB and 0.26, respectively, in clear water, the PSNR and SSIM of our method image reach 15.67 dB and 0.45. Therefore, the application of network algorithms in our work improves the accuracy of the overall SPI system. Although our method can improve the image quality to some extent, there are limitations, mainly, blur in the resultant image, which will be addressed in the future. Also, we will investigate the approaches suitable for turbid water and will expand the structure of the CS-SRCNN to improve image quality further.

## Figures and Tables

**Table 1:** Comparative analysis of different recovery methods with a sampling rate of 18.3% and scattering condition (the signal to noise ratio is 10 dB).

	“Lena”				“Cameraman”			
	SRCNN	CS	Li	Our	SRCNN	CS	Li	Our
PSNR	21.92	18.83	20.47	24.03	21.83	18.48	20.30	23.57
SSIM	0.56	0.32	0.37	0.68	0.57	0.30	0.31	0.59

**Table 2:** The respective peak signal to noise ratio (PSNR) and structural similarity index (SSIM) for different measurements (letter “G”).

	M = 100	M = 200	M = 300
PSNR	9.02	10.17	11.59
SSIM	0.10	0.13	0.26
V	0.24	0.27	0.29

**Table 3:** The respective PSNR and SSIM for different measurements (object “+”).

	M = 100	M = 200	M = 300
PSNR	10.25	11.58	12.78
SSIM	0.23	0.31	0.42
V	0.27	0.29	0.31

**Table 4:** The respective PSNR and SSIM for different methods (the letter “G”).

	SPI Image	SRCNN Image	Our Method Image
PSNR	11.59	12.07	15.67
SSIM	0.26	0.32	0.45
V	0.29	0.30	0.33

**Table 5:** The respective PSNR and SSIM for different methods (object “+”).

	SPI Image	SRCNN Image	Our Method Image
PSNR	12.89	13.21	15.32
SSIM	0.23	0.25	0.36
V	0.31	0.31	0.33

## Data Availability

Data sharing not applicable.
